# Data integration in biological research: an overview

**DOI:** 10.1186/s40709-015-0032-5

**Published:** 2015-09-02

**Authors:** Vasileios Lapatas, Michalis Stefanidakis, Rafael C. Jimenez, Allegra Via, Maria Victoria Schneider

**Affiliations:** Department of Informatics, Ionian University, 7 Tsirigoti Square, Corfu, 49100 Greece; ELIXIR, Wellcome Trust Genome Campus, Hinxton, CB10 1SD UK; Biocomputing Group, Sapienza University, Piazzale Aldo Moro 5, Rome, 00185 Italy; 361° Division, The Genome Analysis Centre, Norwich Research Park, Norwich, NR4 7UH UK

**Keywords:** Data integration, Standards, Bioinformatics, Data driven, Open sciences

## Abstract

Data sharing, integration and annotation are essential to ensure the reproducibility of the analysis and interpretation of the experimental findings. Often these activities are perceived as a role that bioinformaticians and computer scientists have to take with no or little input from the experimental biologist. On the contrary, biological researchers, being the producers and often the end users of such data, have a big role in enabling biological data integration. The quality and usefulness of data integration depend on the existence and adoption of standards, shared formats, and mechanisms that are suitable for biological researchers to submit and annotate the data, so it can be easily searchable, conveniently linked and consequently used for further biological analysis and discovery. Here, we provide background on what is data integration from a computational science point of view, how it has been applied to biological research, which key aspects contributed to its success and future directions.

## Introduction

Data driven biological research has made data integration strategies crucial for the advancements and discovery in a plethora of fields (e.g. genomics, proteomics, metabolomics, environmental sciences, clinical research to name a few) [1–6]. Technically, solutions for data integration have been developed and applied in both corporate and academic sectors. When it comes to biological research, there are different interpretations and levels of data integration people seem to consider [7–14], ranging from genomic data to protein-protein interactions.

Together with data production, there is no doubt that data management, storage and consequently retrieval, analysis and interpretation are at the core of any biological research project. Moreover, the ability to have access to the actual data sets used in a particular study is often crucial for reproducibility and expansion of such study, hence the emphasis in recent years on Open Science and the various initiatives associated [15–21]. Noticeably, in biological research, the difficulties associated with data integration have only expanded with the advent of high throughput technologies [3, 22, 23]. Anyone working with Next Generation Sequencing (NGS) faces challenges associated with a variety of aspects this type of data brings, one of the major being: the volume of the data [24, 25].

Here, we refer to data integration as the computational solution allowing users, from end user (GUI) to power users (API), to fetch data from different sources, combine, manipulate and re-analyse them as well as being able to create new datasets and share these again with the scientific community.

With this definition in mind, it is clear that data integration solutions are imperative for the advancement of research in biological sciences as well as the mechanisms to make such processes traceable, shareable hence “integrable” [26–28]. Here, we provide an overview of the strategies most commonly adopted by the biological research community, current challenges and future directions.

### Key concepts and terminology

Data integration should not just rely on software engineers and computational scientists, but needs to be driven by the actual users whose communities need to define, adopt and use standards, ontologies and annotation best practice. Therefore, it is particularly important for the biological research community to get acquainted with the conceptual basis of data integration, its limitations, challenges and actual terminology.

In order to familiarise the experimental biology community of readers, in Table 1 we present key concepts, definitions and terms used by bioinformaticians and computer scientists.

**Table 1:** Terminology

Schema	A structured and “queryable” way of storing data
Database	A single or collection of schemata
Sources	A number of databases that contain data. Data that reside in each source can either duplicate and/or complement data from other sources
Data Integration	The process of combining data that reside in different sources, to provide users with a unified view of such data
Data Standards	Agreements on representation, format, and definition for common data
Data Formats	A structured way to represent data and metadata in a file
Data Warehousing	Model for integrating data where the data from different sources reside on a central repository (aka data warehouse)
Federated Databases	Model for integrating data where the data reside on the original sources and users are provided with a unified view of the data based on mapping mechanisms of the information
Linked Data	The network of interlinked data that is available on the web. It is used to automatically share semantically rich information and represents the biggest attempt to convert significant amounts of human knowledge across all fields in a computer readable format
Ontology	A structured way of describing data, often presented in a computer-readable format. In bioinformatics, ontologies are sets of unambiguous, universally agreed terms used to describe biological phenomena and “entities”, their properties and their relationships
lled Vocabulary	A collection of terms for describing a certain domain of interest
Unique Identifier	A unique representation for a biological entity (molecule, organism, ontology term, etc.). Usually an alphanumeric string that is used to refer to this entity and distinguishes it from others (much like ID or passport number in humans).
Metadata	Data describing data, i.e., additional information (e.g., a comment, explanation, attributes, etc.) for a specific biological entity or process. As an example, in the context of an ontology, this is used to specify significant properties of the ontology
Annotation	The process of attaching relevant information (metadata) to a raw biological entity
Automatic Annotation	Automatic means that the annotation is being done by computer software (often by transferring information from a source to another). This is a way of producing a large amount of metadata
Manual Annotation	As opposed to automatic annotation, manual means that an actual individual does it
GUI	Graphical User Interface. Is the way that a user interacts with a computer by using graphical icons and visual indicators such as buttons, forms etc. In the scope of this paper we are using the term GUI to refer to interfaces that allow biologists to search/read/edit integrated biological data
API	Application Programming Interface. Set of tool and protocols that a power user can use in order to automatically gain access to functionality and/or data that have been developed/gathered by another individual/organisation
UX	User eXperience. The process of improving user satisfaction by focusing on the usability of a given product.
Visualisation Tools	Applications that help biologists view the data in a more human-friendly way (e.g., Cytoscape for visualising complex networks) like 3D or graph representations of the data

## Review

In computational sciences the theoretical frameworks for data integration have been classified into two major categories namely “eager” and “lazy” [29, 30]. The difference between the two approaches is the way the data get integrated. In the eager approach (warehousing), the data are being copied over to a global schema and stored in a central data warehouse; whereas in the lazy approach the data reside in distributed sources and are integrated on demand based on a global schema used to map the data between sources.

Each of the two main categories of data integration has to deal with its own challenges in order to provide the user with a unified view of the data. In the eager approach, researchers face challenges to keep data updated and consistent, and protect the global schema from having corrupted data [31, 32]. In the lazy approach, data are queried at sources and the scientific community is trying to find ways of improving the answering query process [33–38] and source completeness [36, 37, 39, 40]. Which approach should be used and when depends on amount of data, who owns them and the existing infrastructure.

**Fig. 1:**
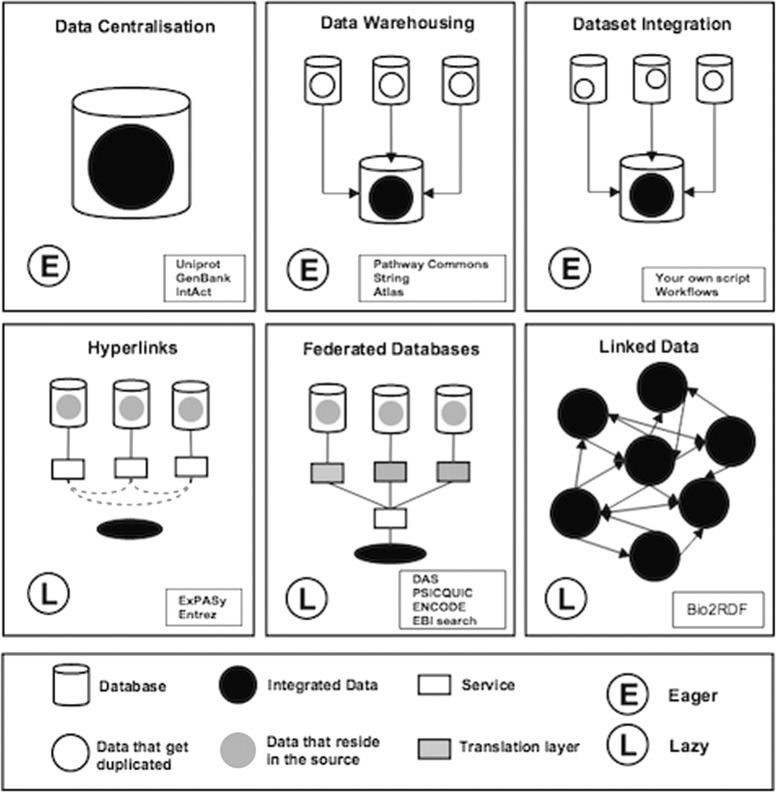
Data integration methodologies. This figure illustrates six major types of data integration methodologies in biology

In biology we see a diversity of implementations across these two approaches being used at a variety of levels and forms like data centralisation, federated databases [41, 42] and linked data [43]. Figure 1 shows the most common schemata used to integrate data in biology.

UniProt [44] and GenBank [45] are examples of centralised resources (Fig. 1-Data Centralisation), whereas Pathway commons [46] collects pathways from different databases and stores them to a shared repository that can be used to query and analyse pathway information (Fig. 1-Data Warehousing). Datasets integration can also be made by in-house workflows accessing distributed databases and downloading data to a local repository (Fig. 1-Dataset Integration). ExPASy [47] is the SIB Bioinformatics Resource Portal through which the user can access databases and tools in different areas of life science (Fig. 1-Hyperlinks). Database links are crucial for interoperability and several efforts have been done in this context [48]. Regarding the federated database model (Fig. 1-Federated Databases), the Distributed Annotation System (DAS) [49] represents a valuable example. DAS is a client-server system used to integrate and display in a single view annotation data on biological sequences residing over multiple distant servers. In this case, a translation layer is needed to achieve data integration among heterogeneous databases. There are various ways to do this but in general it refers to ways to transform the data from the database to a common format so they can be interpreted in the same way from a mapping service. As for the linked data integration (Fig. 1-Linked Data), the services offered are graphical interfaces (GUI) that provide the user with hyperlinks connecting related data from multiple data providers in a large network of Linked Data. BIO2RDF [43] is an example of such integration system.

Data integration in biological research has its challenges associated to a variety of factors such as standards adoption or easy conversion between data/file formats [2].

**Fig. 2:**
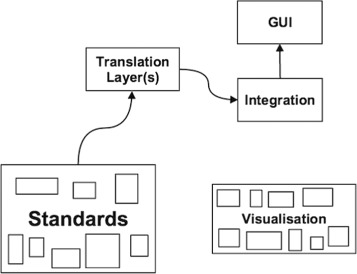
Current state. This figure illustrates a simplified view of the current state of biological data and tools

Figure 2 illustrates a simplified schematic view of the current state of biological research data integration components. Various attempts to integrate the data rely on translation layers that, by applying agreed standards, transform the data in a unified format in order to integrate them. In other words, different formats for the same type of data (e.g. NGS) need to be “translated” into a unified format by applying shared rules. On top of the integration layer, there are various GUIs that make it possible to utilise (download, analyse, represent, etc) the integrated data. Furthermore, there is a myriad of resources and visualisation tools generated that fail to comply with standards and/or are not compatible with each other [50] On the other hand, controlled vocabularies and ontologies to ease data integration are available for an increasing number of biological domain areas. Some of them can be found at the websites of the OBO (Open Biological and Biomedical Ontologies) foundry [51], the NCBO (National Center for Biomedical Ontology) BioPortal [52], and the OLS (Ontology Lookup Service). One successful example is the XML-based proteomic standards defined by the HUPO-PSI (Human Proteome Organisation-Proteomics Standards Initiative) consortium (see Table 2). The rest of the paper will discuss key aspects of standards: ontologies, data formats, identifiers, reporting guidelines, consortiums and standard initiatives which will be followed by a section on visualisation.

**Table 2:** List of data standards initiatives

Acronym	Name	Goal	URL	PMID
OBO	The Open Biological and	Establish a set of principles for ontology	http://www.obofoundry.org	17989687
	Biomedical Ontologies	development to create a suite of orthogonal		
		interoperable reference ontologies in		
		the biomedical domain		
CDISC	Clinical data interchange	Establish standards to support the acquisition,	http://www.cdisc.org	23833735
	standards consortium	exchange, submission and archive of		
		clinical research data and metadata		
HUPO-PSI	Human Proteome Organisation-	Defines community standards for data	http://www.psidev.info	16901219
	Proteomics Standards Initiative	representation in proteomics to facilitate		
		data comparison, exchange and verification		
GAGH	Global Alliance for Genomics	Create interoperable approaches to catalyze	http://genomicsandhealth.org/	24896853
	and Health	projects that will help unlock the great		
		potential of genomic data		
COMBINE	Computational Modeling	Coordinate the development of the various	http://co.mbine.org/	25759811
	in Biology	community standards and formats for		
		computational models		
MSI	Metabolomics Standards	Define community-agreed reporting	http://msi-workgroups.sourceforge.net	17687353
	Initiative	standards, which provided a clear description		
		of the biological system studied and		
		all components of metabolomics studies		
RDA	Research Data Alliance	Builds the social and technical bridges that	https://rd-alliance.org	
		enable open sharing of data across multiple		
		scientific disciplines		

### Standards

As mentioned above, one of the most important factors for the biological field to thrive is to standardise the data. In computational science a similar problem was encountered for the web and specifically with the way that browsers parse web pages. This was solved by agreeing on W3C standards [53] so that all the browsers are forced to comply otherwise they may result in poor user experience and they risk losing market share.

In biology there are many different ways of representing similar data and this makes the data harder to be integrated and processed to obtain unified views of such data. Gene naming is an example of poor uniformity in data representation. Despite full guidelines were issued in 1979 to adopt gene nomenclature standards (see [54]), an assortment of alternate names is still in use across the scientific literature and databases, posing a challenge to data sharing. When it comes to biological research, it is crucial to create (when non existing), adopt and implement standards. Without these it is (nearly) impossible to achieve data integration [55, 56].

So what do we mean by standards? Standards can be defined as an agreed compliant term or structure to represent a biological entity. Entities are all types of units of biological information. For example we use T, G, A, C as a standard way to refer to the nucleotides that make the DNA, and aa (for amino acids) represented usually by one letter, and consequently, a string of letters to represent a DNA or protein sequence. However, a protein might be known in the scientific literature and referred by researchers by a variety of names, synonyms and abbreviations.

So, which standards exist, who defines them and how are these working? Lots of standard initiatives and efforts seem to exist, sometimes redundant, often non driven by the end users communities. It is out of the scope of this paper (and probably a never ending exercise) to review all of them, which do proliferate but not necessarily in harmonising ways. A snapshot of the variety of standards for metadata can be found at the DCC website [57] and BioSharing [58] as an example of the point we are making. Table 2 reports a list of standard initiatives along with their primary goal, URL and key reference in the omics field.

Standards facilitate data re-use. They make data sharing easier, saving overheads and losses of time in data loading, conversion, getting systems to work properly with data. They help overcome interoperability difficulties across different data formats, architectures, and naming conventions, and at infrastructure level, enabling access systems to work together [59–62]. Absence of standards means substantial loss of productivity and less data available to researchers [63].

**Fig. 3:**
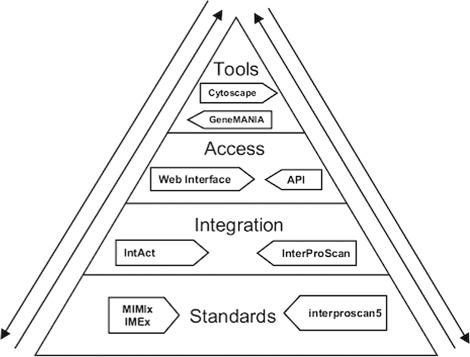
Ideal state. This figure illustrates a simplified view of an ideal state of biological data and tools

Figure 3 illustrates a schematic view of an ideal state of biological research data integration components. This figure emphasises on the importance of standards that is the base of all the top layers of the infrastructure. Without solid foundations, it is very difficult to build and maintain robust tools for the layers above. The arrows point out that the data can be used across all layers and this can go both ways. For example, in an ideal state, all biological data would be integrated from various databases across the world and biologists will be able to use a GUI to locate the entity of their interest. Then, they can use a visualisation tool to have a better representation of the entity by using the same data previously identified through the GUI (like a unique identifier). Furthermore, the biologist will be in a position to annotate or edit the data directly from the visualisation tool, which in turn will be able to commit the changes to the integrated service and from then on go all the way down the pyramid until the data in the proper database get edited and annotated.

Standards are therefore key to the data sharing process since they describe the norms which should be adopted to facilitate interchange and inter-working of information, processes, objects and software. Thus data resources play a major role not just in data management, integration, access, and preservation, but also for providing adequate support to research communities.

#### Ontologies

Ontologies have been proliferating in biological research, and their importance underlined several times [64–67] also in the specific context of data integration [68]. In order to bring some coordination and consolidation to the proliferation of ontologies across the biological and biomedical research fields, The Open Biological and Biomedical Ontologies (OBO) got together. OBO is a collaborative experiment involving developers of science-based ontologies who are establishing a set of principles for ontology development with the goal of creating a suite of orthogonal interoperable reference ontologies in the biomedical domain. Biological researchers can get involved and provide feedback by getting into the discussion fora OBO provides. Currently there are ten OBO foundry ontologies and more than 120 candidate ontologies or other ontologies of interest [51].

These efforts need the direct involvement of the actual biologists when it comes to the adoption and implementation of using such ontologies, ensuring these are known and disseminated across communities. Other important initiatives are, the NCBO (National Center for Biomedical Ontology) BioPortal [69, 70], and the OLS (Ontology Lookup Service) [71].

With a set of unique common compliant standards in place, it will be possible to create tools to integrate the data on the web using an existing infrastructure like linked data. This will enable querying multiple sources without having to re-invent integration techniques for the integration of each source. As an example, one of the efforts currently trying to attempt this is Bio2RDF [43]. This is a major effort to integrate biological data using the linked data infrastructure. So far there are no tools that can utilise these data directly but they are mainly accessible via complex queries or low level GUIs.

### Formats

Data formats are the concrete way we structure and represent biological information in a file. They are particularly relevant to those who deal with large amount of information such that generated by high throughput experiments. Indeed, a scientist interested in a single or a few genes at a time may extract information about them by manually “parsing” the literature or free-text (i.e. non formatted) documents. The need for storing biological data in formatted files arose from the need for using computers to analyse them. The amounts of genomics and proteomics data, which cannot be manually analysed element by element, are exponentially increasing and the adoption of commonly agreed formats to represent them in computer readable files is nowadays of utter importance. Historically, the scarcity of well structured data standards and schemas, caused the flourishing of many different formats even to represent the same type of data despite the adoption of standards in file formats would be essential to data exchange and integration. Funnily, the Roslin Bioinformatics Law’s First Law declaims: “The first step in developing a new genetic analysis algorithm is to decide how to make the input data file format different from all pre-existing analysis data file formats” [72].

For the benefit of data integration though, it would be ideal to have well-structured data across few basic formats that would be easily computer readable and therefore easily integrated. In the specific case of NGS data, the lag between the emerging high-throughput screening technologies and the adjusting of the scientific community to settle on a standard format, means time and effort spent on converting raw files across multiple sequencing platforms to make these compatible [73]. Currently, in NGS there are no really “standards” that people adhere to, but a set of commonly used formats (FASTA/Q, SAM, VCF, GFF/GTF, etc.). There are descriptor standards like MIGS [74], but these might not be generally adopted. More in general, today an exhaustive “atlas” of the formats used in bioinformatics cannot be found on the Internet. One partial list is available at http://genome.ucsc.edu/FAQ/FAQformat.html and the description of many formats can be found in the online forum BioStar [75].

A good format needs to take into account the data themselves (for example the DNA sequence of a gene) and the so called metadata, i.e. additional information describing the data (e.g. gene name, taxonomy information, cross reference to other resources, etc.) and has to adopt strategies (“tricks”) to make metadata unequivocally distinguishable from data by a computer program. This goal is achieved in different ways by different bioinformatics resources, resulting in the large number of formats we observe today. However, despite the large variety of computer readable formats, we realised that the most commonly used ones are ascribable to four main different classes: 1) tables 2) FASTA-like 3) GenBank-like 4) tag-structured. Table 3 reports examples for each of these classes.

**Table 3:** Mostly commonly used data formats in bioinformatics

Data format class	General data-	Nucleotide sequence	Protein sequence	Structural	Sequence	Other data
	interchange formats	data	data	data	alignment	types (PPI, etc)
Tabl	CSV, TSV	BED; GFF	GFF, Uniprot-GFF	PSF(D), MMCIF(D)	SAM(D)	
FASTA-like		FASTA; FASTQ	FASTA, PIR		SAM(M)	Wig
GenBank-like		GenBank; EMBL	Uniprot-TEXT	PDB, PSF(M), MMCIF(D)	CLUSTAL, MSF,	
					PHYLIP(D)	
Tag-structured	HTML; XML; JSON	SBOL-XML	Uniprot-XML;			PSI MI-XML;
			Uniprot-RDF/XML			PSI-PAR

D = data; M = metadata. Formats appearing in more than one class are a mixture of classes

**Fig. 4:**
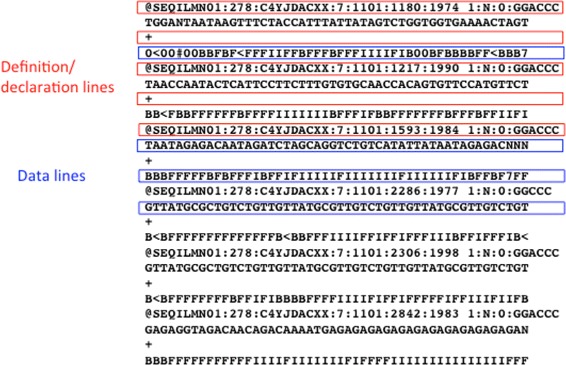
Selected parts of a FASTQ file. In this format declaration lines start with two different characters (“@” and “+”) corresponding to different data types (the raw sequence and the sequence quality values, respectively)

**Fig. 5:**
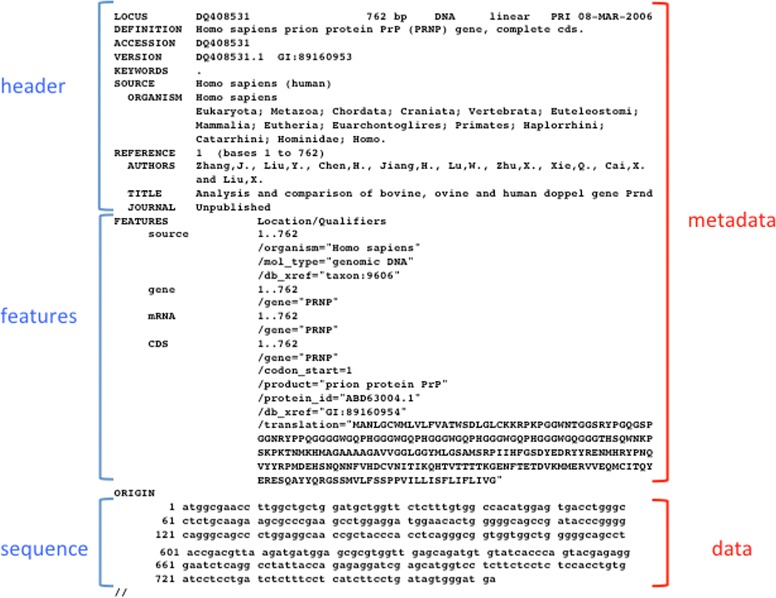
Selected parts of the GenBank entry DQ408531. The complete entry can be found at http://www.ncbi. nlm.nih.gov/nuccore/DQ408531

**Fig. 6:**
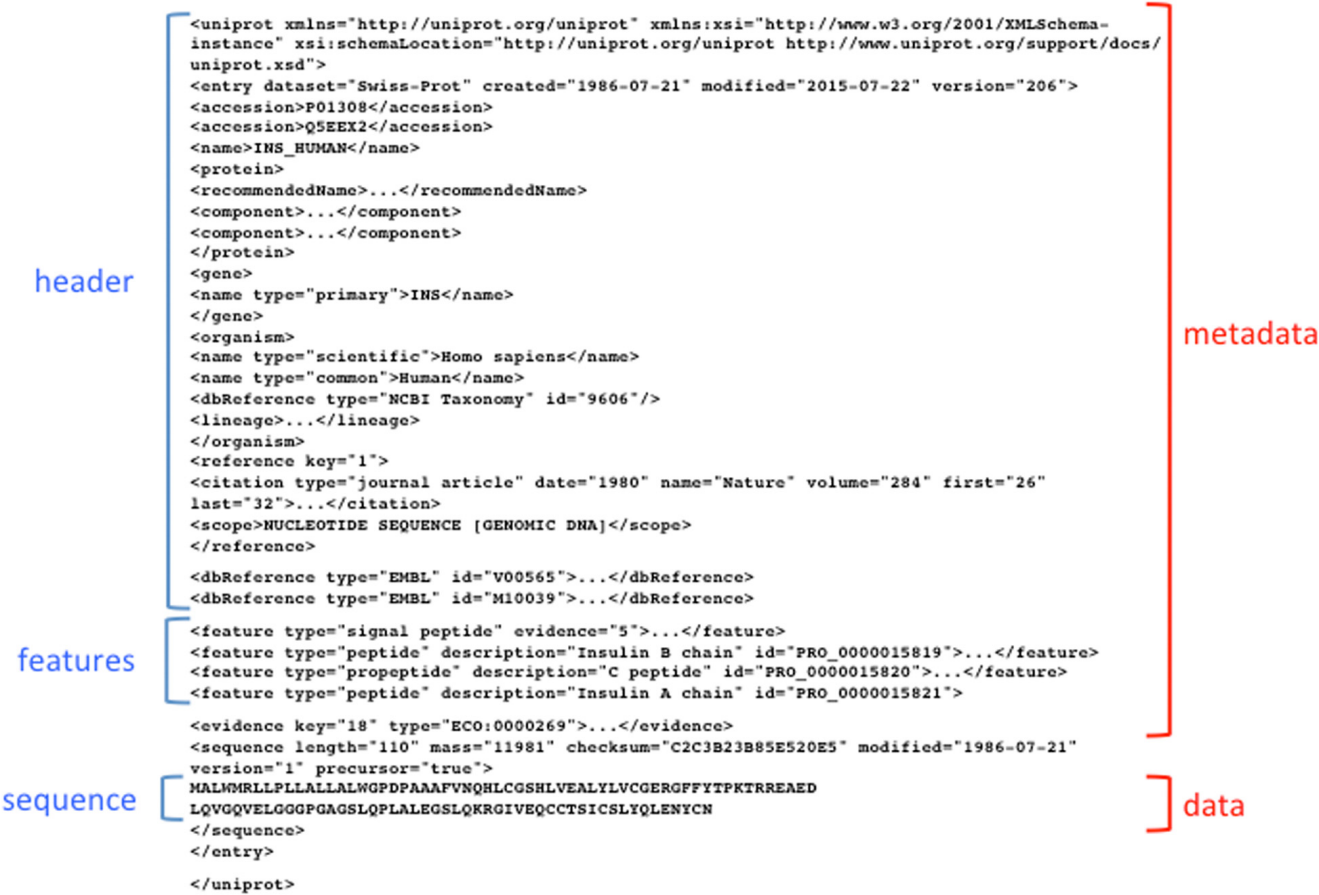
Selected parts of the Uniprot entry P01308 in XML format - The complete entry can be found at http://www.uniprot.org/uniprot/P01308.xml

In table formats, data are organised in a table in which the columns are separated by tabs, commas, pipes, etc., depending on the source generating the file. FASTA-like files utilise, for each data record, one or more “definition” or “declaration lines”, which contain metadata information or specify the content of the following lines. Definition/declaration lines usually start with a special character or keyword in the first position of the line - a “ >” in FASTA files or a “@” in fastq or SAM files - followed by lines containing the data themselves (Fig. 4). In some cases, declaration lines may be interspersed with data lines. This format is mostly used for sequence data. In the GenBank-like format, each line starts with an identifier that specifies the content of the line (Fig. 5). Tag-structured formatting uses “tags” (“ <”, “ >”, “{”, “}”, etc.) to make data and metadata recognisable (Fig. 6) with high specificity. Tag-structured text files, especially XML and JSON, are being increasingly employed as data interchange formats between different programming languages.

**Fig. 7:**
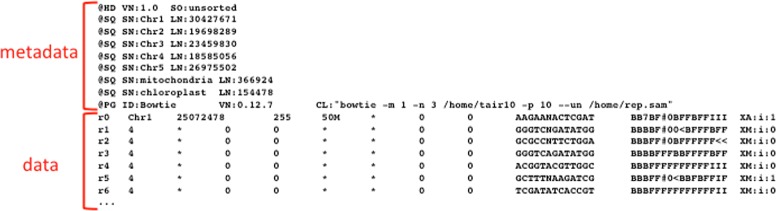
Selected parts of a SAM file

There are also examples of data files using different representations for data and metadata. This means that two or more format classes may be used in the same data file. An example is represented by SAM files, which contain both GenBank-like lines (for the metadata) and table columns (for the data) as shown in Fig. 7.

Should any of these four data representation classes be preferred over the others? Despite we observe an increasing use of XML and some authors propose to adopt XML for biological data interchange between databases and other sources of data [76], we believe that there is not an ultimate answer. There are text formats that better suit some specific kind of data and specific computational requirements and purposes. For example, it is difficult to imagine how macromolecule X-ray or NMR coordinates and related annotation, currently stored in PDB files, could fit into the FASTA-like format. On the other hand, if one has to parse big sequence files, the FASTA format, with a single line annotation, will cause them to have a smaller size than differently formatted files and will allow parsing them with just a few lines of code. Notice that some formats (e.g. SAM) can be compressed into a binary version (BAM) for intensive data processing.

Therefore, we believe that the solution is not to urge scientists to conform to a unique “optimal” format but rather to identify a few operational formats and make database and tool developers aware of the importance of sticking to them.

For integration purposes, the scientific community of database and tool developers has begun to adopt some good practices in data file formatting. One example is represented by the FGED Society (http://fged.org/) formed at a meeting on Microarray Gene Expression Databases (EBI, Hinxton, 1999) with the goal, amongst the others, of facilitating the adoption of standards for DNA microarrays and gene expression data representation. We believe, however, that further efforts should be made in order to achieve a more robust and systematic policy in all the areas where data sharing is essential to utilise these data to make new discoveries and the progress of science possible.

The community of scientists concerned by data sharing and integration, including us, should make the effort of 1) compiling a complete and structured (i.e. organised by data type and purpose) list of the currently available formats with their description and 2) developing guidelines and recommendations for the adoption of standards in file formatting, also discussing which data types fit into each different text format and the related performance implications. This list and the guidelines, which might be integrated in a resource such as BioSharing should encourage database and tool developers to present information in a way that a computer program can parse it, suggest that they avoid inventing new computer readable formats but rather comply with one of the existing ones, and only accept new data, for storage purposes, that meet certain formatting criteria. Such guidelines should be ambitious and forward-looking enough to also advice scientists in both academia and industry to keep in mind data representation in developing high throughput technologies and their information services.

The development of converters translating formats in a unified form should be promoted as well. This would actually make it possible to combine the data across all the formats. A rather isolated example of data format translation is represented by the PRIDE Converter [77], which makes it easy to translate a large variety of input formats into the unique XML [76, 78] format for proteomic data submission to the PRIDE repository [79]. The PRIDE Converter was designed to be suitable for both small and large data submissions and has a very intuitive GUI also for wet-lab scientists without a strong bioinformatics background or informatics support. Format translation faces problems especially with not well-structured data that cannot be translated properly in a computer readable format and therefore rely on human manipulation of the data in order to verify the correctness of the transformation. In the case of NGS data, we rely on tools for conversion between next generation sequencing data formats, such as NGS-FC (http://sourceforge.net/projects/ngsformaterconv/), to ensure each tool in a workflow can work with the right format.

#### Identifiers

An identifier is a unique representation of a given data entry [80, 81]. For example the Universal Protein Database (UniProt) uses a “unique identifier” to refer to a protein entity which cannot be used in any other case, thus ensuring no redundancy and one agreed unique term that unequivocally identifies a given protein [82].

In biological research a variety of data repositories exist and each of them is using its own implementation for generating unique identifiers. As an example, for the same protein, UniProt uses the identifier Q9Y6N8 whereas Ensembl [83] is referring to it as ENSP00000264463 and RefSeq [84] as NP_006718.2. If all the researchers could use a single unique identifier to refer to a given protein across their publications and work, data integration would be a step ahead of its current state.

An effort to help with the discoverability of the identifiers and assist the researcher with knowledge on how to query data across databases has be done from identifiers.org [85]. This is a registry that facilitates the discovery of resources in life sciences and allows to decouple the identification of records by the physical locations on the web where they can be retrieved.

Many biological concepts are described in several databases using different identifiers. To facilitate discoverability and integration, databases have their data entries cross-referenced with external entries using identifiers. This enables users to find a data entry like a protein in UniProt and then find the same biological concept described in other databases (ie. RefSeq) and gather more relevant data about the same entry. Several initiatives like PICR [86] or the “DAVID ID conversion tool” [87] provide mapping of such identifiers. It will be beneficial if such service gets integrated in the major bioinformatics databases.

Some organised efforts including distributed resources like IMEx [88] are very well organised and, though the independent databases that are part of the consortium like IntAct [81], MINT [89] and DIP [90] use their own identifiers, all their entries get assigned a unique IMEx identifier issued by a central authority. The IMEx identifier is assigned to a single biological entity with the purpose of being reused across databases/systems and always link to the same entity regardless the system. The IMEx Central repository coordinates curation effort, assigns identifiers and facilitates the exchange of completed records on molecular interaction data between the IMEx Consortium partners.

Approaches like these can increase discoverability and shareability of data and even enable publications and scientific studies to use a single identifier to refer to a given entity. This entity could be easily traced and further studied by their audience. With an infrastructure like this in place, it will be possible to enforce researchers to submit the unique identifier of the biological entity that they are studying on their research papers. This is happening already for nucleotide sequence data where researchers have to submit newly obtained/sequenced entities to one of the three major sequencing databases [91] and refer to it in the paper. Most of other data types can be used in publications without such requirement. This also extends to entire datasets.

#### Reporting guidelines

Huge steps have been achieved by the creation and adoption of clear recommended guidelines when it comes to depositing and disseminating data and datasets [92–95]. Such guidelines are often the result of several discussions (years of discussions in some occasions) in a field where data efforts for sharing have been maturing. The specification of several standards in life science include documentation and examples of how to use them, but many initiatives additionally include guidelines to agree on what minimum or recommended information should be provided when describing data. Minimum information guidelines have been very popular to ensure that data can be easily interpreted and that results derived from their analysis can be independently verified. These guidelines tend to concentrate on defining the content and structure of the necessary information rather than the technical format for capturing it. A key landmark in the development of guidelines of minimun information in this area comes from the “Minimum Information about a Biomedical or Biological Investigation” (MIBBI) [93].

It is crucial to have a place where such efforts are listed and shared in order to ensure redundancy is avoided. As an example of reporting guidelines we mention here the efforts done in the topic of protein-protein interactions. Currently we see two reporting guidelines: MIMIx [96] and IMEx [88]. A key project that is contributing in this area and where one can look for as well as add “reporting guidelines” is the Registry of guidelines in biosharing.org [58, 97].

As we have seen, there are different formats when it comes to data files, and these will always evolve according to the needs of the communities as well as the nature of the data and associated technologies. For example, a format that contains 20 fields for which one researcher might have a subset of information versus another that might opt for prioritising a different set. It is clear that having a minimum agreed set of fields that all comply to report using standards is crucial for data integration and reusability across such data. Similarly, other fields might be crucial and informative to a specific set of users. These can be adopted at the level of recommended. For example a protein-protein interaction database wants to capture domain specific information about interactions versus another one that is not interested in such aspect. One also might have optional fields, for those that want to annotate and enrich further the data record with metadata. Doing this in a standard manner means again allowing future reusability and expansion for others to adopt and exchange, integrate data based on this level of information.

#### Consortiums and standards initiatives

There are several initiatives coordinating the development of community standards to facilitate data comparison, exchange and verification in bioinformatics. Some of this initiatives are community initiatives or consortia like COMBINE [98], PSI [99], GAGH [100], INSDC [101], proteomeXchange [102], IMEx [88], BioPax [103] involved in the development of standards in one specific biological domain. Some other community initiatives like RDA are more generic with a potential application in different scientific domains.

Some strategic efforts supported by major service providers and national governments like ELIXIR [104], BBMRI [105], BD2K [106] are also involved in the development of standards in life sciences. Projects supported by specific grants like BioMedBridges [107], BioSHaRE [108] do also contribute to this cause but their duration is normally bound to the duration of the grant. All these initiatives play a major role in achieving consensus and agreements which facilitates the development and adoptions of standards.

In biological research, molecular biology has been the field ahead in terms of such efforts and the associated bioinformatics applications. One can only imagine the work yet to be done, learning from existing efforts and initiatives as described here in the field of ecology, biodiversity, marine biology and so on. Examples of large scale efforts that need to talk to each other and ideally apply best practice when it comes to creating an infrastructure that fosters data integration are LifeWatch [109] and ISBE [110].

#### Visualisation

There is a variety of visualisation tools, but often each tool requires a different file format and the task of feeding back the discovered data is not trivial [111, 112]. The field of visualisation has its own challenges given the increasing quantity of data, the integration of heterogeneous data and the need for tools that allow representing multiple aspects of the data (e.g. multiple connections between nodes with diverse biological meanings [113, 114]). There is a myriad of visualisation and analysis tools, ever proliferating, with each tool providing specific features that address different aspects (e.g. genome browsers [115–119]). In 2008 Pavlopoulus et al published a wish list for visualisation of biological data which still remains valid [120].

Data integration principles are fundamental in providing tools that are user friendly and allow the end users (biologists) to focus their efforts on the actual study of the data instead of being lost in the process of looking for the data they need by querying multiple databases that appear to provide inconsistent results between them. The field of systems biology *per se* brought substantial advances in visualisations since the ability to analyse and interpret interactions, networks and pathways relies often in the ability of visualising these accurately [120].

Overcoming some of the challenges associated with visualisation relies on better standards adoption and improvement in annotation and metadata. This is clearly a two directional effort: bottom up, where data and datasets are annotated and stored following a common set of standards, this extends to the data formats as well as a top down level of standards and adoption of compatible formats and output files that allow comparisons and integrations of results [121–123].

Historically, many domains within biology have relied on visualisation as a way to represent the biological information thus creating what are now considered standards in their domains. Plenty of examples can be found in the areas of phylogenetics [124] and pathways [125, 126]. The advent of next generation sequencing brought genomics as a domain were significant effort has been put to develop new visualisation techniques to represent sequences, alignments, expression patterns and ultimately entire genomes [127–130]. However, biological researchers might lack an understanding and awareness about the range of visualisation techniques available and which is the most appropriate visual representation [131, 132].

An increased dialogue between the computational scientists involved in the creation and development of such tools with the end users (aka the biologists), would be beneficial for the entire community and we hope this paper is one step towards such outcome. Efforts in this direction are also on the way and we cite here the BiVi initiative (http://bivi.co/), which is addressing several challenges in the realm of visualisation as well as trying to reduce the gap between the biology, computational sciences and developers of bioinformatics tools. BiVi has grouped many of the most notable visualisation tools produced by biologists and developers across seven domains (though some of the tools cover more than one of these) and provides information as to their provenance, current status and links to websites (http://bivi.co/visualisations). Other community efforts in this area are VizBI (http://vizbi.org/), SciVis (http://scivis.itn.liu.se/) and CoVis (http://www.iwr.uni-heidelberg.de/groups/CoVis/).

It would be impossible for us to list the plethora of visualisation tools developed and used in biological research, hence we provide an overview in Table 4 of some of the most common visualisations tools in the area of “Interaction Network Visualisation” to illustrate the variety and types of resources available for one area.

**Table 4:** Common visualisation tools in the area of “Interaction Network Visualisation”

Name of resource	What it does	URL
BicOverlapper	Visualisation of biclusters combined with profile plots and heat maps	http://vis.usal.es/bicoverlapper/
BiGGEsTS	Heat map-based bicluster visualisation	http://tinyurl.com/BiGGEsTS
Brain Explorer	Visualisation of 3D transcription data in the central nervous system	http://tinyurl.com/brainExplorer
Data Matrix Viewer	Simple profile plot visualisation; supports Gaggle	http://gaggle.systemsbiology.net/
EXPANDER	Heat maps, scatter plots and profile plots of cluster averages	http://acgt.cs.tau.ac.il/expander
GENESIS	Analysis suite; offers several interactive visualisations	http://genome.tugraz.at/
geWorkbench	Modular suite; heat maps, dendrograms, profile and scatter plots	http://tinyurl.com/geWorkbench
Hierarchical Clustering Explorer	Linked heat map, profile and scatter plots; systematic exploration	http://tinyurl.com/HCExplorer
Java TreeView	Linked heat maps, karyoscopes, sequence alignments, scatter plots	http://jtreeview.sourceforge.net/
Mayday	Modular suite; many linked visualisations; enhanced heat map113	http://tinyurl.com/maydaywp
MultiExperiment Viewer	Analysis suite; heat maps, dendrograms, profile and scatter plots	http://www.tm4.org/
PointCloudXplore	Visualisation of 3D transcription data in Drosophila embryos	http://tinyurl.com/PointCloudXplore
TimeSearcher	Exploration and analysis of time series; advanced profile plots	http://tinyurl.com/timesearcher
R/BioConductor Geneplotter	Karyoscope-style plots and other visualisations	http://www.bioconductor.org/
GenePattern	Modular analysis platform; several visualisation modules available	http://tinyurl.com/GenePatt
Cytoscape	Open source software platform for visualizing molecular interaction networks and biological pathways and integrating these networks with annotations, gene expression profiles and other state data	http://www.cytoscape.org/index.html

There are also well known and generally adopted analysis suites that also provide visualisation tools as part of their repertoire of resources such as Galaxy [133], Cytoscape [134, 135], Ondex [136], iPlant Collaborative [137], Bioconductor [138]. Other important efforts derive from initiatives that are working towards unlocking the actual visualisations, in other words going from the visualisation to the data and datasets. This is important not only for reproducibility but also to allow access for data and their integration with other data/datasets. A very interesting resource is Utopia Docs [139, 140], a free PDF reader that connects the static content of scientific articles to the dynamic world of online content. This resources allows the user to interact directly with curated database entries; play with molecular structures; edit sequence and alignment data; even plot and export tabular data. Another totally different but relevant initiative in the world of visualisation is BIOJS, that aims to provide open-source library of JavaScript components to visualise biological data. BIOJS vision is that every online biological dataset in the world should be visualised with BIOJS tools (http://biojs.net/) [141, 142].

## Conclusion

Data heterogeneity is one of the biggest challenges in biological data integration. This could be solved with standardising the data structures that are being used. Biologists should get more involved with the aspects described here and working with bioinformaticians and computational scientists to achieve uniformity of their data. With this issue resolved, integration of biological data will greatly boost biological research and the field will gain a more robust structure: computational scientists will be responsible for maintaining and improving the infrastructure of the data; bioinformaticians will be able to build upon this infrastructure; biologists will be able to do research with advanced tools without the overhead of getting acquainted with complex topics of database management and programming tools.
